# Trends in smoking prevalence and attitude toward tobacco control among members of the JCA in 2004–2017

**DOI:** 10.1111/cas.15289

**Published:** 2022-02-16

**Authors:** Yuri Ito, Kota Katanoda, Seiichiro Yamamoto, Nobuyuki Hamajima, Yumiko Mochizuki, Keitaro Matsuo

**Affiliations:** ^1^ 13010 Department of Medical Statistics Research & Development Center Osaka Medical and Pharmaceutical University Osaka Japan; ^2^ 576721 National Cancer Center Institute for Cancer Control Tokyo Japan; ^3^ 576721 Shizuoka Graduate University of Public Health Shizuoka Japan; ^4^ Department of Healthcare Administration Nagoya University Graduate School of Medicine Nagoya Japan; ^5^ Shinmachi Clinic & Health Care Center Tokyo Japan; ^6^ Division of Cancer Epidemiology and Prevention Department of Preventive Medicine Aichi Cancer Center Research Institute Nagoya Japan; ^7^ Department of Cancer Epidemiology Nagoya University Graduate School of Medicine Nagoya Japan

## DISCLOSURE

There are no financial or other relations that could lead to a conflict of interest regarding this study. NH and KM are current Editorial Board members of Cancer Science.

Smoking is the single most preventable cause of death in the world.^1^ In Japan, 130,000 people die from active smoking every year,^2^ and about 15,000 people die from secondhand smoke exposure.^3^ Tobacco control is the most effective strategy to prevent smoking‐related cancer, and political action regarding regulation and legislation for smoking has been taken throughout the world.^4^ As a result, the prevalence of smoking has decreased substantially in many countries, including Japan.^5^

In April 2020, the Revised Health Promotion Act and the Tokyo Metropolitan Ordinance to Prevent Exposure to Secondhand Smoke were enacted. These followed the comprehensive evidence report of smoking risk including secondhand smoke “A White Paper on Tobacco,” which was summarized by an expert committee of the Ministry of Health, Labour and Welfare, and an increase in public opinion in favor of preventing secondhand smoke exposure before the Tokyo 2020 Olympic and Paralympic Games. Before the changes to the smoking‐related law, the Japanese Cancer Association (JCA) also revised its Declaration Against Smoking and Tobacco Use in 2016 while starting to discuss the content of the Revised Health Promotion Act. The declaration was originally published in 2003. Recently, we revised our declaration (English version: supplementary document), stressing on banning studies funded by tobacco‐related industries from the society's meetings and journal.

To monitor JCA members’ smoking status and awareness of tobacco control, we conducted surveys in 2004, 2006, 2010,^6^, ^7^ and 2017. The former three surveys were based on physical mail for randomly sampled members, and the most recent one was conducted using the internet survey system “SurveyMonkey” for all the members who had registered e‐mail addresses. This survey was approved as an activity of the Committee Against Smoking at a board meeting of the JCA.

Of a total of 12,500 JCA members, 3867 responded to the 2017 survey (response rate: 30.9%). More young JCA members responded than older ones. Although the number of responding members was higher, the response rate was lower than in the previous surveys (662/1146 = 57.8% in 2004, 920/1523 = 60.8% in 2006, and 1511/3184 = 47.4% in 2010). The proportion of current smokers was 4.0% (4.8% in men and 0.7% in women), a slight decrease, and was still low in 2017. The proportion of never‐smokers increased from 52.3% in 2004 to 69.8% in 2017 (Table 1), which was related to the increase in new young members. The prevalence of smoking among JCA members was lower than in the general population,^8^ and trends of never‐smokers were similar to those among the young generation generally. Compared with the prevalence of smoking found in other medical associations’ surveys (the survey of the Japan Medical Association in 2016: 10.9% in men and 2.4% in women), the prevalence of smoking among JCA members was quite low.

**TABLE 1:** Smoking prevalence according to background characteristics among survey responders

Characteristics	2017 (*n* = 3867)			2010 (*n* = 1511)			2006 (*n* = 920)			2004 (*n* = 662)		
	Current smoker	Former smoker	Never‐smoker	Current smoker	Former smoker	Never‐smoker	Current smoker	Former smoker	Never‐smoker	Current smoker	Former smoker	Never‐smoker
Total	155 (4.0%)	1,012 (26.2%)	2,700 (69.8%)	80 (5.3%)	508 (33.6%)	923 (61.1%)	83 (9.0%)	273 (29.7%)	564 (61.3%)	39 (5.9%)	277 (41.8%)	346 (52.3%)
Sex												
Male	150 (4.8%)	960 (30.6%)	2,025 (64.6%)	71 (6.2%)	438 (38.1%)	642 (55.8%)	74 (10.3%)	237 (33.0%)	408 (56.7%)	35 (6.4%)	241 (44.1%)	270 (49.5%)
Female	5 (0.7%)	45 (6.3%)	660 (93.0%)	3 (1.4%)	14 (6.3%)	205 (92.3%)	1 (0.9%)	9 (7.8%)	106 (91.4%)	0	2 (6.5%)	29 (93.5%)
Age												
20–39	50 (4.2%)	162 (13.5%)	992 (82.4%)	20 (4.8%)	78 (18.9%)	315 (76.3%)	21 (8.7%)	40 (16.5%)	181 (74.8%)	7 (9.3%)	8 (10.7%)	60 (80.0%)
40–59	87 (3.9%)	634 (28.7%)	1,488 (67.4%)	47 (5.4%)	313 (35.8%)	514 (58.8%)	59 (10.7%)	173 (31.3%)	320 (58.0%)	20 (5.8%)	150 (43.7%)	173 (50.4%)
≥60	18 (4.0%)	216 (47.6%)	220 (48.5%)	13 (5.8%)	117 (52.2%)	94 (42.0%)	3 (2.4%)	60 (47.6%)	63 (50.0%)	12 (4.9%)	119 (48.8%)	113 (46.3%)

Note

Not all subjects responded to questions regarding sex or age.

Awareness of the revised version of the JCA’s Declaration Against Smoking and Tobacco Use was monitored in the 2017 survey. Only 10% of responders knew the declaration in detail. Never‐smokers reported less awareness of the declaration compared with former/current smokers. Older members were more aware of the declaration than younger members (Table 2). The results indicate the need to make efforts to inform members about the revised version of the JCA’s Declaration Against Smoking and Tobacco Use. The Committee Against Smoking has started activities to make summaries of recommended papers about smoking as a cancer risk available for members of the JCA and for the general population on our website (https://www.jca.gr.jp/hi.html).

**TABLE 2:** Awareness of the Japanese Cancer Association's (JCA) Declaration Against Smoking in the 2017 survey

Characteristics	2017 (*n* = 3867)			*P*‐value
	Do not know	Know declaration, but not in detail	Know declaration in detail	
Total	1322 (34.2%)	2144 (55.4%)	401 (10.4%)	
Smoking status				<0.001
Current smoker	35 (22.6%)	101 (65.2%)	19 (12.3%)	
Former smoker	296 (29.3%)	612 (60.5%)	104 (10.3%)	
Never‐smoker	991 (36.7%)	1,431 (53.0%)	278 (10.3%)	
Sex				0.001
Male	1036 (33.1%)	1,747 (55.7%)	352 (11.2%)	
Female	276 (38.9%)	385 (54.2%)	49 (6.9%)	
Age				<0.001
20–39	573 (47.6%)	552 (45.9%)	79 (6.6%)	
40–59	678 (30.7%)	1295 (58.6%)	236 (10.7%)	
≥60	71 (15.6%)	297 (65.4%)	86 (18.9%)	

Note

Not all subjects responded to questions regarding sex or age.

We also asked JCA members whether or not they support each of the 11 items of the declaration (Table 3). More than 90% of responders supported item 1 (promotion of studies to the tobacco control), item 5 (nonsmoking conference), item 6 (promotion of education to prevent harmful smoking), item 7 (smoking cessation in clinical/health checkup settings), and item 11 (preventing smoking by minors). However, surprisingly, 16.7% of responders did not support and only 60.5% supported item 2, which is about strictly banning JCA members from receiving funding from tobacco‐related industries. We analyzed item 2, on which opinions were divided, in detail. By smoking status, 42.9% of current smokers did not support item 2, while 19.1% of former smokers and 14.3% of never‐smokers did not support the item. Based on research field, 19.6% of basic researchers did not support the item, while 14.4% of clinical researchers and 16.5% of social researchers did not support the item (Table 4). Respondents who did not engage in clinical practice were more likely to disagree with the item than responders who did (18.9% vs. 14.8%). Following cross‐tabulation by age categories and research field, only 47.6% of 20–39‐year old responders in basic research supported item 2; this proportion was noticeably lower than other combinations of age groups and research fields (Table 5). We also analyzed determinant factors for disagreement with item 2 using a logistic regression model. Current smokers and basic researchers (or no engagement in clinical practice) tended to disagree with item 2 (Table 6). Most items (except 1, 7, 10) had less support from younger responders (data not shown). As the smoking rate among young people has decreased recently, young members may be less interested in the tobacco control issue. We need to make efforts to share the importance of tobacco control by involving young members of the JCA in activities against tobacco use.

**TABLE 3:** Support for each item in the Japanese Cancer Association's (JCA) Declaration Against Smoking and Tobacco Use in the 2017 survey

		Support	Neutral	Don't Support
Item 1	We promote studies on harmful effects of smoking, development of effective smoking cessation, and other issues relevant to our country's tobacco control policies	3580 (92.8%)	206 (5.3%)	71 (1.8%)
Item 2	Members of the society will not receive funding from tobacco‐related industries or institutions run by such industries. Studies funded by such bodies will be banned from presentation in the society's meetings and submissions to the society's journals	2332 (60.5%)	879 (22.8%)	642 (16.7%)
Item 3	We will set an example to the society by promoting a complete ban of smoking in all premises of institutions to which our members are affiliated. Members who smoke will actively make an effort to quit smoking	3293 (85.6%)	358 (9.3%)	198 (5.1%)
Item 4	We will seize every opportunity to advocate the harms of tobacco to patients and the wider community to promote non‐smoking	3351 (87.0%)	380 (9.9%)	119 (3.1%)
Item 5	All meetings held by the Japanese Cancer Association, including the annual meeting, and the facilities where they will take place will be non‐smoking	3559 (92.4%)	186 (4.8%)	107 (2.8%)
Item 6	We promote education on harmful effects of tobacco and smoking prevention measures for minors	3762 (97.8%)	68 (1.8%)	18 (0.5%)
Item 7	We promote smoking cessation to all smokers and encourage treatment and support for those who wish to quit smoking at medical institutions and at health checkups	3,513 (91.2%)	250 (6.5%)	88 (2.3%)
Item 8	We promote a complete ban of smoking in public places, including restaurants and workplace, in order to prevent harmful effects of secondhand smoke exposure	3275 (85.0%)	346 (9.0%)	230 (6.0%)
Item 9	We will reinforce regulations on tobacco‐related advertisements and vending machines as well as the health warnings	3060 (79.5%)	575 (15.0%)	212 (5.5%)
Item 10	We will support raising the prices of tobacco to the same level as western developed countries and using a part of the increase in tax revenue for the promotion of tobacco regulation	3261 (84.8%)	399 (10.4%)	187 (4.9%)
Item 11	We strive to prevent smoking by minors and effects of secondhand smoke on non‐smokers	3770 (98.1%)	55 (1.4%)	18 (0.5%)

Note

Not all subjects responded to questions regarding each item.

**TABLE 4:** Characteristics of subjects regarding item 2 in the declaration

	Support	Neutral	Do not support	*P*‐value
Smoking status				<0.001
Current smoker	56 (36.4%)	32 (20.8%)	66 (42.9%)	
Former smoker	605 (60.0%)	211 (20.9%)	192 (19.1%)	
Never‐smoker	1671 (62.1%)	636 (23.6%)	384 (14.3%)	
Sex				<0.001
Male	1903 (60.9%)	661 (21.2%)	559 (17.9%)	
Female	414 (58.5%)	214 (30.2%)	80 (11.3%)	
Age				<0.001
20–39	680 (56.7%)	319 (26.6%)	201 (16.8%)	
40–59	1334 (60.6%)	487 (22.1%)	380 (17.3%)	
60+	318 (70.4%)	73 (16.2%)	61 (13.5%)	
Research field				<0.001
Clinical	1269 (66.1%)	373 (19.4%)	277 (14.4%)	
Basic	854 (54.9%)	396 (25.5%)	305 (19.6%)	
Social	54 (68.4%)	12 (15.2%)	13 (16.5%)	
Other	148 (51.8%)	95 (33.2%)	43 (15.0%)	
Engagement in clinical practice				<0.001
Yes	1,365 (65.2%)	419 (20.0%)	309 (14.8%)	
No	965 (57.9%)	460 (26.2%)	333 (18.9%)	

Note

Not all subjects responded to questions regarding each item.

**TABLE 5:** Cross‐tabulation of attitude to item 2 in the declaration according to age and research field

		Support	Neutral	Do not support	*P*‐value
Age	Research field				
20–39	Clinical	391 (67.7%)	122 (21.1%)	65 (11.3%)	<0.001
	Basic	242 (47.6%)	155 (30.5%)	111 (21.9%)	
	Social	4 (30.8%)	6 (46.2%)	3 (23.1%)	
	Others	42 (43.8%)	34 (35.4%)	20 (20.8%)	
40–59	Clinical	730 (63.9%)	226 (19.8%)	187 (16.4%)	0.001
	Basic	486 (56.5%)	165 (19.2%)	210 (24.4%)	
	Social	36 (75.0%)	6 (12.5%)	6 (12.5%)	
	Others	77 (54.6%)	44 (31.2%)	20 (14.2%)	
≥60	Clinical	148 (74.8%)	25 (12.6%)	25 (12.6%)	0.002
	Basic	126 (67.7%)	31 (16.7%)	29 (15.6%)	
	Social	14 (77.8%)	0 (0%)	4 (22.2%)	
	Other	29 (59.2%)	17 (34.7%)	3 (6.1%)	

Note

Not all subjects responded to questions regarding each item.

**TABLE 6:** Determinant factors for opposition to item 2 in the declaration using logistic regression model

	Univariate analysis				Multivariate analysis 1				Multivariate analysis 2				Multivariate analysis 3			
	OR	95% CI		*P*‐value	OR	95% CI		*P*‐value	OR	95% CI		*P*‐value	OR	95% CI		*P*‐value
Sex																
Male	1				1				1				1			
Female	0.59	0.46	0.75	<.001	0.57	0.44	0.74	<.001	0.57	0.44	0.74	<.001	0.57	0.44	0.73	<.001
Age																
20–39	1				1				1				1			
40–59	1.04	0.86	1.25	0.706	1.00	0.82	1.21	0.972	1.01	0.83	1.22	0.934	1.00	0.83	1.22	0.982
60+	0.77	0.57	1.06	0.105	0.71	0.52	0.98	0.035	0.72	0.52	0.98	0.04	0.72	0.52	0.98	0.038
Smoking status																
Never‐ or former smoker	1				1				1				1			
Current smoker	4.04	2.90	5.62	<.001	3.77	2.70	5.27	<.001	3.73	2.67	5.21	<.001	3.76	2.69	5.26	<.001
research field																
Clinical	1				1								1			
Basic	1.44	1.21	1.72	<.001	1.55	1.29	1.86	<.001					1.41	1.07	1.86	0.016
Social or other	1.11	0.82	1.51	0.495	1.17	0.86	1.60	0.32					1.06	0.72	1.55	0.776
Engagement in clinical practice																
Yes	1								1				1			
No	1.32	1.11	1.56	0.001					1.42	1.19	1.69	<.001	1.14	0.86	1.49	0.363

Abbreviations: CI, confidence interval; OR, odds ratio.

Tobacco industry–funded research institutions (e.g., the Smoking Research Foundation and the Foundation for a Smoke‐Free World) gave funds to a wide range of research areas including basic research. The Smoking Research Foundation, which was funded by the Japan Tobacco Inc., started funding young researchers in 2016. Four of the five most recently funded research projects for young cancer researchers were basic research. Some researchers in the JCA might have received funding from tobacco industries previously. For young researchers, funding opportunities are essential but raise a conflict of interest (COI). Japan Tobacco Inc. used to have a scholarship program for high school students to study at university. We need a fair system to support young researchers and students financially. The mandatory system to report COIs, which started in 2011 in the JCA, can help to maintain the transparency of our research activities against cancer. But the policy depends on researchers’ voluntary report of all potential COI.^9^

In the current survey in 2017 and previous surveys in 2006 and 2010, we asked questions about smoking cessation treatment (Table S1). When we limited responders to those who were working at a clinical setting, most provided or introduced smoking cessation treatment for patients who needed it (over 95% in all periods; Table S2). The problem of insurance coverage for patients (i.e., insurance companies did not include young or moderate smokers in their target population) had been solved by 2017 (30%–40% in 2006 and 2010 vs. 0% in 2017; Table S3). Since 2006, smoking cessation treatment has been covered by medical insurance. In 2016, the target of the smoking cessation treatment covered by medical insurance was expanded to young patients and/or moderate smokers. Our results showed a trend of increased cessation treatment; however, JCA members still feel some barriers such as limited time for consultation and lack of education on smoking cessation treatment. As an academic association committed to preventing cancer, we need to provide more opportunities for clinicians among the JCA members to learn about smoking cessation programs in collaboration with other academic associations.

Recently, use of new tobacco products including heat‐not‐burn tobacco has become prevalent among young people.^10^, ^11^ Tobacco industries advertise the lower risk of the product compared with cigarettes; however, long‐term risks and other potentially fatal risks are unknown.^12^ To fight against a new enemy of tobacco control, our academic society should boost activities to study the risk of new tobacco products. In 2020, the JCA’s Committee Against Smoking held a special session “The Era of New Types of Tobacco: Science and Social Impact” in the annual JCA meeting in Hiroshima to share the latest research and discuss future problems. In the next survey for JCA members, we will monitor the prevalence of new tobacco product use and attitudes toward them. As a leader in the cancer research community, the JCA should take action to deal with the control of new tobacco products globally.

## Supporting information

Table S1‐S3

Supplementary Material
