# Supplementary material for: Trends in smoking prevalence and attitude toward tobacco control among members of the JCA in 2004–2017
Source: Cancer Sci. 2022 Feb 16;113(4):1542–7. doi: 10.1111/cas.15289 (PMC8990729; doi:10.1111/cas.15289)
Supplement: Supplementary file 1 — Table S1‐S3 [file CAS-113-1542-s002.docx]

Supplementary Table 1. Response of all other questions by smoking status

|  |  | Total | | Current Smokers | | Former-smokers | | Never-smokers | |
| --- | --- | --- | --- | --- | --- | --- | --- | --- | --- |
|  |  | N=3,867 | | N=155 | | N=1,012 | | N=2,700 | |
| Q8. What do you think about patients smoking? | | | | | | | | | |
|  | Should not smoke, because they are sick. | 1,808 | (46.9%) | 21 | (13.6%) | 443 | (44.2%) | 1,344 | (49.9%) |
|  | It depends on their disease. | 389 | (10.1%) | 29 | (18.8%) | 111 | (11.1%) | 249 | ( 9.2%) |
|  | It is their choice. | 1,455 | (37.8%) | 99 | (64.3%) | 391 | (39.0%) | 965 | (35.8%) |
|  | I don't know. | 199 | ( 5.2%) | 5 | ( 3.2%) | 58 | ( 5.8%) | 136 | ( 5.0%) |
| Q9. Do you provide treatment of smoking cessation for patients if they need it? | | | | | | | | | |
|  | Yes, I do directly or I will introduce a doctor who can provide the treatment. | 2,304 | (59.9%) | 85 | (55.6%) | 644 | (64.0%) | 1,575 | (58.6%) |
|  | No, I don't. | 30 | ( 0.8%) | 3 | ( 2.0%) | 10 | ( 1.0%) | 17 | ( 0.6%) |
|  | I don't know. | 1,423 | (37.0%) | 56 | (36.6%) | 332 | (33.0%) | 1,035 | (38.5%) |
|  | I don't work in a clinical setting. | 92 | ( 2.4%) | 9 | ( 5.9%) | 21 | ( 2.1%) | 62 | ( 2.3%) |
| Q10. What are the barriers to providing treatment for smoking cessation? | |  |  |  |  |  |  |  |  |
|  | 1. Limited time for consultation | 1,159 | (30.0%) | 40 | (25.8%) | 317 | (31.3%) | 802 | (29.7%) |
|  | 2. Limited target for insured treatment | 0 | ( 0.0%) | 0 | ( 0.0%) | 0 | ( 0.0%) | 0 | ( 0.0%) |
|  | 3. No need for cancer patients | 122 | ( 3.2%) | 17 | (11.0%) | 30 | ( 3.0%) | 75 | ( 2.8%) |
|  | 4. Have not been trained in smoking cessation methods | 860 | (22.2%) | 36 | (23.2%) | 235 | (23.2%) | 589 | (21.8%) |
|  | 5. Useless because it is not successful | 57 | ( 1.5%) | 6 | ( 3.9%) | 9 | ( 0.9%) | 42 | ( 1.6%) |
|  | 6. Patient refused treatment the first time | 892 | (23.1%) | 27 | (17.4%) | 246 | (24.3%) | 619 | (22.9%) |
|  | 7. Hospital does not support the treatment | 119 | ( 3.1%) | 4 | ( 2.6%) | 28 | ( 2.8%) | 87 | ( 3.2%) |
| Q11. What do you think about the revision of the Occupational Health and Safety Act, which promotes measures against secondhand smoke exposure in the workplace by the obligation to provide a separate smoking area and to provide a subsidy to faciliate this? | | | | | | | | | |
|  | Satisfied | 1,653 | (43.1%) | 89 | (57.8%) | 413 | (41.1%) | 1,151 | (43.0%) |
|  | Not satisfied | 940 | (24.5%) | 10 | ( 6.5%) | 260 | (25.9%) | 670 | (25.0%) |
|  | Neither | 1,243 | (32.4%) | 55 | (35.7%) | 331 | (33.0%) | 857 | (32.0%) |
| Q12. What is your opinion of the tobacco control measures under consideration by the government? | | | | | | | | | |
| (1) A law banning secondhand smoke exposure will be established in the host city (Tokyo) of the Olympic game in 2020. | | | | | | | | | |
|  | Support | 3,403 | (88.4%) | 82 | (53.2%) | 876 | (87.2%) | 2,445 | (90.9%) |
|  | Don't support | 144 | ( 3.7%) | 32 | (20.8%) | 44 | ( 4.4%) | 68 | ( 2.5%) |
|  | Neither | 301 | ( 7.8%) | 40 | (26.0%) | 85 | ( 8.5%) | 176 | ( 6.5%) |
| (2) Removal of limitation to treat patients whose Brinkman Index is over 200 using medical insurance. | | | | | | | | | |
|  | Support | 2,511 | (65.5%) | 83 | (53.9%) | 636 | (63.7%) | 1,792 | (66.8%) |
|  | Don't support | 147 | ( 3.8%) | 13 | ( 8.4%) | 49 | ( 4.9%) | 85 | ( 3.2%) |
|  | Neither | 1,178 | (30.7%) | 58 | (37.7%) | 314 | (31.4%) | 806 | (30.0%) |
| (3) Increasing tobacco tax to reduce the consumption of tobacco and improve health | | | | | | | | | |
|  | Support | 3,182 | (83.0%) | 66 | (42.9%) | 810 | (81.0%) | 2,306 | (86.1%) |
|  | Don't support | 198 | ( 5.2%) | 56 | (36.4%) | 62 | ( 6.2%) | 80 | ( 3.0%) |
|  | Neither | 453 | (11.8%) | 32 | (20.8%) | 128 | (12.8%) | 293 | (10.9%) |
| Q13. (If you agreed to increase tobacco tax in Q12(3)) What should be the price of one pack of cigarettes? | | | | | | | | | |
|  | 500 yen | 165 | (5.2%) | 18 | (27.3%) | 51 | (6.3%) | 96 | (4.2%) |
|  | 600 yen | 80 | (2.5%) | 3 | (4.5%) | 18 | (2.2%) | 59 | (2.6%) |
|  | 700 yen | 154 | (4.8%) | 4 | (6.1%) | 38 | (4.7%) | 112 | (4.9%) |
|  | 800 yen | 391 | (12.3%) | 7 | (10.6%) | 103 | (12.7%) | 281 | (12.2%) |
|  | 900 yen | 88 | (2.8%) | 1 | (1.5%) | 25 | (3.1%) | 62 | (2.7%) |
|  | Over 1,000 yen | 2,419 | (76.0%) | 51 | (77.3%) | 611 | (75.4%) | 1,757 | (76.2%) |
| Q14. In Japan, the government is considering decreasing the legal age for drinking alcohol and smoking tobacco. On the other hand, many other countries are increasing the legal age. In Tasmania State, Australia, the law of "tobacco-free generation" for the birth cohort after 2000 is under consideration. Singapore, Scotland, and Ireland are following. What do you think of the political trends to prevent smoking among the younger generation? | | | | | | | | | |
|  | Support | 3,154 | (82.0%) | 94 | (61.0%) | 815 | (80.9%) | 2,245 | (83.6%) |
|  | Don't support | 285 | ( 7.4%) | 21 | (13.6%) | 66 | ( 6.6%) | 198 | ( 7.4%) |
|  | Don't know | 408 | (10.6%) | 39 | (25.3%) | 126 | (12.5%) | 243 | ( 9.0%) |
| Q16. What is your opinion of the tobacco control activities by the Committee Against Smoking in the JCA? | | | | | | | | | |
| (1) The process of revising the JCA's Declaration Against Smoking and the PR activities. | | | | | | | | | |
|  | Support | 3,376 | (88.1%) | 97 | (62.6%) | 859 | (85.8%) | 2,420 | (90.4%) |
|  | Don't support | 56 | ( 1.5%) | 9 | ( 5.8%) | 21 | ( 2.1%) | 26 | ( 1.0%) |
|  | Neither | 400 | (10.4%) | 49 | (31.6%) | 121 | (12.1%) | 230 | ( 8.6%) |
| (2) The survey about smoking status and attitude towards tobacco control among members of the JCA | | | | | | | | | |
|  | Support | 3,153 | (82.4%) | 92 | (59.7%) | 796 | (80.0%) | 2,265 | (84.6%) |
|  | Don't support | 131 | ( 3.4%) | 12 | ( 7.8%) | 43 | ( 4.3%) | 76 | ( 2.8%) |
|  | Neither | 541 | (14.1%) | 50 | (32.5%) | 156 | (15.7%) | 335 | (12.5%) |
| (3) Collecting information about tobacco control and publishing the information, which includes the statement for the general population and government | | | | | | | | | |
|  | Support | 3,522 | (92.2%) | 119 | (78.3%) | 901 | (90.5%) | 2,502 | (93.7%) |
|  | Don't support | 43 | ( 1.1%) | 6 | ( 3.9%) | 14 | ( 1.4%) | 23 | ( 0.9%) |
|  | Neither | 254 | ( 6.7%) | 27 | (17.8%) | 81 | ( 8.1%) | 146 | ( 5.5%) |

Supplementary Table 2. Attitudes toward smoking cessation treatment for cancer patients by smoking status (limited to subjects who are clinicians)

|  |  | Total | | Current smokers | | Former-smokers | | Never-smokers | | p-value |
| --- | --- | --- | --- | --- | --- | --- | --- | --- | --- | --- |
|  |  | N=2,041 |  | N=78 |  | N=579 |  | N=1,384 |  |  |
| Q8. What do you think about patients smoking? | |  |  |  |  |  |  |  |  |  |
|  | Should not smoke, because they are sick. | 1,062 | (52.2%) | 12 | (15.6%) | 286 | (49.7%) | 764 | (55.3%) | <0.001 |
|  | It depends on their disease. | 220 | (10.8%) | 15 | (19.5%) | 67 | (11.7%) | 138 | (10.0%) |  |
|  | It is their choice. | 698 | (34.3%) | 50 | (64.9%) | 199 | (34.6%) | 449 | (32.5%) |  |
|  | I don't know. | 54 | ( 2.7%) | 0 | ( 0.0%) | 23 | ( 4.0%) | 31 | ( 2.2%) |  |
| Q9. Do you provide treatment of smoking cessation for patients if they need? | |  |  |  |  |  |  |  |  |  |
|  | Yes, I do directly or I will introduce a doctor who can provide the treatment. | 1,962 | (96.1%) | 70 | (89.7%) | 560 | (96.7%) | 1,332 | (96.2%) | 0.012 |
|  | No, I don't. | 27 | ( 1.3%) | 3 | ( 3.8%) | 10 | ( 1.7%) | 14 | ( 1.0%) |  |
|  | I don't know. | 52 | ( 2.5%) | 5 | ( 6.4%) | 9 | ( 1.6%) | 38 | ( 2.7%) |  |
| Q10. What are the barriers to providing treatment for smoking cessation? | |  |  |  |  |  |  |  |  |  |
|  | 1. Limited time for consultation | 1,020 | (50.0%) | 28 | (35.9%) | 279 | (48.2%) | 713 | (51.5%) | 0.016 |
|  | 2. Limited target for the insured treatment | 0 | ( 0.0%) | 0 | ( 0.0%) | 0 | ( 0.0%) | 0 | ( 0.0%) |  |
|  | 3. No need for cancer patients | 91 | ( 4.5%) | 15 | (19.2%) | 21 | ( 3.6%) | 55 | ( 4.0%) | <0.001 |
|  | 4. Have not been trained in smoking cessation methods | 713 | (34.9%) | 28 | (35.9%) | 201 | (34.7%) | 484 | (35.0%) | 0.98 |
|  | 5. Useless because it is not successful | 38 | ( 1.9%) | 2 | ( 2.6%) | 5 | ( 0.9%) | 31 | ( 2.2%) | 0.11 |
|  | 6. Patient refused the first time | 731 | (35.8%) | 19 | (24.4%) | 215 | (37.1%) | 497 | (35.9%) | 0.086 |
|  | 7. Hospital does not support the treatment | 93 | ( 4.6%) | 2 | ( 2.6%) | 24 | ( 4.1%) | 67 | ( 4.8%) | 0.55 |

Supplementary Table 3. Trends in attitudes toward smoking cessation treatment for cancer patients

|  |  | 2006 |  | 2010 |  | 2017 |  | p-value |
| --- | --- | --- | --- | --- | --- | --- | --- | --- |
|  |  | N=694 |  | N=485 |  | N=2,041 |  |  |
| Q8. What do you think about patients smoking? | |  |  |  |  |  |  |  |
|  | Should not smoke, because they are sick. | 339 | (49.2%) | 258 | (53.5%) | 1,062 | (52.2%) | 0.003 |
|  | It depends on their disease. | 117 | (17.0%) | 64 | (13.3%) | 220 | (10.8%) |  |
|  | It is their choice. | 218 | (31.6%) | 149 | (30.9%) | 698 | (34.3%) |  |
|  | I don't know. | 15 | ( 2.2%) | 11 | ( 2.3%) | 54 | ( 2.7%) |  |
| Q9. Do you provide treatment of smoking cessation for patients if they need? | |  |  |  |  |  |  |  |
|  | Yes, I do directly or I will introduce a doctor who can provide the treatment. | 663 | (95.5%) | 472 | (97.3%) | 1,962 | (96.1%) | 0.36 |
|  | No, I don't. | 7 | ( 1.0%) | 5 | ( 1.0%) | 27 | ( 1.3%) |  |
|  | I don't know. | 24 | ( 3.5%) | 8 | ( 1.6%) | 52 | ( 2.5%) |  |
| Q10. What are the barriers to providing treatment for smoking cessation? | |  |  |  |  |  |  |  |
|  | 1. Limited time for consultation | 384 | (55.3%) | 290 | (59.8%) | 1,020 | (50.0%) | <0.001 |
|  | 2. Limited target for the insured treatment | 269 | (38.8%) | 143 | (29.5%) | 0 | ( 0.0%) | <0.001 |
|  | 4. Have not been trained in smoking cessation methods | 191 | (27.5%) | 150 | (30.9%) | 713 | (34.9%) | 0.001 |
