# Supplementary material for: Trends in smoking prevalence and attitude toward tobacco control among members of the JCA in 2004–2017
Source: Cancer Sci. 2022 Feb 16;113(4):1542–7. doi: 10.1111/cas.15289 (PMC8990729; doi:10.1111/cas.15289)
Supplement: Supplementary file 2 — Supplementary Material [file CAS-113-1542-s001.docx]

Declaration Against Smoking and Tobacco Use

1. We promote studies on harmful effects of smoking, development of effective smoking cessation, and other issues relevant to our country’s tobacco control policies.

2. Members of the society will not receive funding from tobacco related industries or institutions run by such industries. Studies funded by such bodies will be banned from presentation in the society’s meetings and submissions to the society’s journals.

3. We will set an example to society by promoting a complete ban of smoking in all premises of institutions where our members to which they are affiliated. Members who smoke will actively make an effort to quit smoking.

4. We will seize every opportunity to advocate the harms of tobacco to patients and the wider community to promote non-smoking.

5. All meetings held by Japan Cancer Association, including the annual meeting, and the facilities where they will take place will be non-smoking.

6. We promote education on harmful effects of tobacco and smoking prevention measures for minors.

7. We promote smoking cessation to all smokers and encourage treatment and support for those who wish to quit smoking at medical institutions and at health checkups.

8. We promote a complete ban of smoking in public places, including restaurants and workplace, in order to prevent harmful effects of secondhand smoke exposure.

9. We will reinforce regulations on tobacco related advertisements and vending machines as well as the health warnings.

10. We will support raising the prices of tobacco to the same level as western developed countries and using a part of the increase in tax revenue for the promotion of tobacco regulation.

11. We strive to prevent smoking by minors and effects of secondhand smoke on non-smokers.
